# Internationalization is Necessary, But is it Enough?

**DOI:** 10.5935/abc.20180212

**Published:** 2018-10

**Authors:** Gláucia Maria Moraes de Oliveira, Andrea De Lorenzo, Fernanda Marciano Consolim Colombo, Eduardo Back Sternick, Andréa Araujo Brandão, Sergio Emanuel Kaiser, Alexandre Schaan de Quadros, Renato Abdala Karam Kalil, Christianne Brêtas Vieira Scaramello, Francisco Rafael Martins Laurindo, Ludhmila Abrahão Hajjar

**Affiliations:** 1 Programa de Pós-Graduação em Cardiologia da Universidade Federal do Rio De Janeiro (UFRJ), Rio de Janeiro, RJ - Brazil; 2 Programa de Pós-Graduação em Ciências Cardiovasculares do Instituto Nacional de Cardiologia (INC), Rio de Janeiro, RJ - Brazil; 3 Programa de Pós-Graduação em Medicina da Universidade Nove de Julho (UNINOVE), São Paulo, SP - Brazil; 4 Programa de Pós-Graduação em Ciências da Saúde da Faculdade Ciências Médicas de Minas Gerais, Belo Horizonte, MG - Brazil; 5 Programa de Pós-Graduação em Ciências Médicas Universidade do Estado do Rio de Janeiro (UERJ), Rio de Janeiro, RJ - Brazil; 6 Programa de Pós-Graduação em Fisiopatologia Clínica e Experimental da Universidade Estadual do Rio de Janeiro (UERJ), Rio de Janeiro, RJ - Brazil; 7 Programa de Pós-Graduação do Instituto de Cardiologia do Rio Grande do Sul/Fundação Universitária de Cardiologia (IC/FUC), Porto Alegre, RS - Brazil; 8 Programa de Pós-Graduação em Cardiologia da Universidade de São Paulo (USP), São Paulo, SP - Brazil; 9 Programa de Pós-Graduação em Ciências Cardiovasculares da Universidade Federal Fluminense (UFF), Rio de Janeiro, RJ - Brazil

**Keywords:** Scientifcs Periodicals/internacionalization, International Cooperation, Researcher Performance Evaluation System, Journal Impact Factor, Databases, Bibliographics, Citation Databases

Globalization has left its mark on the 21^st^ century. One of the many ways of defining globalization is as the integration of information, communication, and economy on a worldwide scale, with a direct influence on all levels of higher education. In this manner, the internationalization of postgraduation may be seen as a response to globalization, taking shape in the form of programs and policies put in place by academic institutions and governments in order to increase student and faculty exchanges and to stimulate and strengthen partnerships in research, among other actions. Universities and research centers have, in fact, been practicing these actions for a long time, but they have expanded significantly, particularly during this century.

Various studies^1^^-^^3^ have repeatedly shown that collaborative research that involves authors from multiple institutions and/or countries have an identifiably greater impact than research involving only one group or institution. In Brazil, the internationalization of postgraduation has been highly valued by the Coordination for the Improvement of Higher Education Personnel (CAPES), generating immense efforts on the part of postgraduate programs (PGPs) to achieve the goals defined. In a study conducted with PGPs ranked 6 or 7 by CAPES, Ramos^1^ observed that internationalization in these PGPs encompasses everything from international mobility, international cooperation networks, academic output (international publications, international co-authorships, presentation of academic work in international scientific conferences and meetings), to access to resources through the sharing of research facilities and international funding. In Brazilian PGPs, the most popular internationalization strategies were international mobility of faculty, researchers, and students and international research collaboration, implemented mainly through international cooperation agreements. This study, nevertheless, detected inequalities between institutions in the provision of adequate conditions for internationalization. The availability of financial resources, the existence of regulatory frameworks, and organizational support were considered important requisites for achieving this goal.^1^

Thus, due to the demands of CAPES, the need to internationalize has led to an institutional “race” between PGPs in search of partners, with or without government support, and often in a competitive manner. The strategies adopted by each institution vary in accordance with their “scope” (based on professors and researchers’ contacts or previously established partnerships) and their level of resources and complexity capable of influencing international visibility and competitiveness. This “academic entrepreneurship”^2^ may or may not be considered positive, given that more well known institutions often have a head start in the competition for funds, to the detriment of other academic centers. The existence of national policies that support internationalization, including the publication of national journals, is extremely important, as can be seen in the example of countries that have successfully invested in this form of support.^3^

In order to meet the demands imposed by CAPES, PGPs and medical societies are faced with the challenge of making a joint effort to provide all academic institutions with access to opportunities and to allow the scientific output that originates from these PGPs to be disseminated by their national and international peers. In this context, the Brazilian Society of Cardiology (SBC) has held “Postgraduate Meetings in Cardiovascular Sciences.” The theme of the fourth meeting, in 2018, was “The Internationalization of Brazilian Postgraduate Programs.”

During this meeting, the international guest speaker Professor Fausto J. Pinto, the Director of the Faculty of Medicine of the University of Lisbon, spoke about partnerships between European and Brazilian universities and announced the signing of a recent agreement between the Portuguese institution and the Federal University of Rio de Janeiro’s School of Medicine for the bilateral recognition of diplomas and dual degrees. He emphasized the importance of reinforcing contacts between universities in Brazil and Europe, especially in Portugal, in order to make the dissemination and adaptation of existing models possible by establishing a network of Portuguese-language medical schools as an element to facilitate exchange, in addition to the creation of an “Erasmus-like” program for the Community of Portuguese Language Countries (CPLP).

The visibility of national research is another important aspect of internationalization. It is of the utmost importance to disseminate research carried out by the Brazilian scientific community in the area of cardiovascular disease (CVD), which is recognized as the leading cause of mortality in Brazil and worldwide. The SBC has two journals indexed in SciELO: the *Arquivos Brasileiros de Cardiologia (ABC Cardiol)* and the *International Journal of Cardiovascular Sciences.* The *ABC Cardiol*, which is indexed in the main databases, including ISI Web of Science, Index Medicus, MEDLINE, PubMed Central, EMBASE, Scopus, SciELO and LILACS, obtained an Impact Factor (IF) of 1.318 from Journal Citation Reports (JCR), as well as a B2 rating from the CAPES Qualis System in its most recent evaluation.^4^

The *ABC Cardiol* is Latin America’s main journal for the publication of research in the area of Cardiology and Cardiovascular Sciences and it has an important and increasing degree of internationalization, as more than 20% of its articles are of international origin. It is worth emphasizing that Portuguese language countries (PLCs) have access to the bilingual version of the *ABC Cardiol*, which is published in all Lusophone countries by the Portal of the Federation of Cardiology Societies of Portuguese Language Countries (http://www.fsclp.org), representing approximately 245 million people. In PLCs, the large differences, related mainly to socioeconomic conditions, in the relative impact of CVD burden are noteworthy.^5^ The highest quality of scientific output published in the *ABC Cardiol* continues to originate from Brazilian postgraduate programs, which are increasingly exposed, in the meanwhile, to international competition to find publishing space in the *ABC Cardiol*, added to the lower incentive to publish in this journal as a direct result of its current CAPES ranking. It is also very important to valorize the publication of science and knowledge, which are public goods, in open science journals such as the ABC Cardiol and other journals in the SciELO network.

CAPES values the social involvement of PGPs, with the objective of promoting improvements in the population’s living conditions. However, Brazilian studies focusing on populations with peculiar socioeconomic characteristics rarely receive the interest of the international community; their dissemination would need to be driven by CAPES’ evaluation system in order to strengthen a national exchange network that included PLCs. The innovative scientific contributions that result from PGPs and their mission for civil society are also worth highlighting. In this sense, it would be desirable for this regulatory agency to create a system, mediated by Qualis, for valuing the leading journal in the fight against the CVD epidemic in a manner that makes it possible to share successful experiences in fighting CVD with these countries.

An unprecedented academic revolution is taking place in higher education, leading to a real need for the internationalization of PGPs. Internationalization offers new opportunities for study and research that are limited neither by national boundaries nor by boundaries to knowledge. However, “internationalization is not a goal in itself, but rather a means to accomplish improvements in teaching, research, and innovation,”^6^ as well as to promote the development of a more just and equitable society through improvements in the living conditions of the population; this, upon final analysis, is the main objective of research undertaken by PGPs. The recognition of national research must also occur ethically and meritocratically through the valuing of the means responsible for its dissemination. The growth of Brazilian journals will have to be the fruit of intellectual investment on the part of researchers who produce quality science and internationalization through international partnerships, as well as stimulation and valorization through better rankings in CAPES’ evaluation system.
